# Sublingual sufentanil after orthopaedic and abdominal surgery: long-term outcome and safety

**DOI:** 10.1186/s13741-025-00506-y

**Published:** 2025-02-28

**Authors:** Michael Borck, Jan D. Wandrey, Claudia Spies, Sascha Tafelski

**Affiliations:** 1 Charité–Universitätsmedizin Berlin, corporate member of Freie Universität Berlin, Humboldt-Universität zu Berlin, and Berlin Institute of Health, Charitéplatz 1, Berlin, 10117 Germany; 2 Department of Anaesthesiology and Intensive Care, Campus Charité Mitte and Campus Virchow-Klinikum, Augustenburger Platz 1, Berlin, 13353 Germany; 3 Berlin Institute of Health at Charité–Universitätsmedizin Berlin, BIH Biomedical Innovation Academy, BIH Charité Digital Clinician Scientist Program, Charitéplatz 1, Berlin, 10117 Germany

**Keywords:** Patient-controlled analgesia, Sublingual sufentanil tablet system, Postoperative pain management, Postoperative delirium, Postoperative chronic pain

## Abstract

**Background:**

Acute postoperative pain management often requires opioid treatment with patient-controlled analgesia (PCA). Non-invasive PCA with a sublingual sufentanil tablet system (SSTS) may reduce acute pain sufficiently, but opioids are associated with central nerve system side effects and risk of long-term opioid use postoperatively. The objective of this study was to observe the SSTS to assess the incidence of postoperative chronic pain (PCP) and postoperative delirium (POD).

**Methods:**

This was a longitudinal cohort study based at a university hospital between November 2017 and November 2021. Adults undergoing elective orthopaedic knee or abdominal surgery planned for PCA as postoperative pain management were included. They received the SSTS in addition to a standardised pain medication protocol depending on the surgery they underwent. Exclusion criteria were pregnancy, emergency surgery, concurrent participation in another clinical trial and chronic opioid use before surgery. Patients were followed after surgery in hospital and over 3 and 12 months for pain, cognitive function and side effects.

**Results:**

Altogether *N* = 80 patients were included with SSTS postoperatively. Daily pain experience decreased from pre-operatively 89.2% of patients to 45.7% and 22.5% at 3 and 12 months. None of our patients developed postoperative delirium after surgery. Patients reported high overall satisfaction with SSTS (median 8.0/10 points, IQR 3). However, 51% of patients had difficulties with handling the SSTS and required acute replacement of the authentication tag.

**Conclusions:**

SSTS sufficiently treated acute postoperative pain without incidence of POD and demonstrated good tolerability and overall ease. Postoperative pain improved significantly over time but 22% still reported chronic pain related to surgery. Technical issues with the identification thumb tag limited the feasibility of SSTS.

**Trial registration:**

This prospective longitudinal cohort study was approved by the ethics committee of the Charité Universitätsmedizin Berlin (Ethics committee 2, Campus Charité Virchow Klinikum, EA2/041/17, Prof. Dr. jur. R. Seeland, 21.03.2017) and was registered in the study register (https://clinicaltrials.gov/ct2/show/NCT03133858).

## Background

Optimal treatment of perioperative pain is a key issue in surgery to enhance postoperative recovery and patient satisfaction. Inadequate pain therapy can lead to postoperative complications such as postoperative nausea and vomiting (PONV), inadequate nutrition, lack of mobilisation and an increased risk for postoperative delirium (POD) (Aldecoa et al. 2017). Additionally, long-term sequelae may develop such as postoperative cognitive dysfunction (POCD) or postoperative chronic pain (PCP) (Aldecoa et al. 2017). Especially patients with orthopaedic and abdominal surgery may experience high acute pain postoperatively (Gerbershagen et al. 2013). Individual anxiety or depression modulates pain perception (Kastelik et al. 2019). However, self-efficiency (Shipton and Stuart-Smith 2016; Lovich-Sapola et al. 2015) with postoperative patient-controlled analgesia alleviates pain after surgery (Bandura 1977). Therefore, for effective postoperative analgesia, patients may profit from individualised solutions (Grass 2005) which remain a clinical challenge in resource-restricted environments. In a previous RCT in knee arthroplasty, Kastelik et al. (Kastelik et al. 2019) described a gap in controlling acute postoperative pain on exertion with local infiltration analgesia (LIA) compared with continuous saphenous nerve block. Although both groups achieved comparable mobilisation and overall satisfaction, acute pain exacerbations on exertion raised the question for an optimised perioperative pain management (Kastelik et al. 2019) to enhance postoperative rehabilitation. PCA systems have been well established in providing effective analgesia for patients as they ensure an immediate and by-demand application of an adequate dose of pain medication (Abrolat et al. 2018). Thus, they support active participation in pain control which has been shown to have a positive impact on overall patient satisfaction (Elmallah et al. 2018). The main available PCA systems need intravenous access (PCIA). A main drawback of PCIA is impaired mobilisation due to intravenous lines and potential complications of intravenous opioids (Grass 2005) such as catheter displacement, bleeding or infection (Donk et al. 2018). Addressing these disadvantages, sublingual sufentanil tablet systems (SSTSs) were developed and approved for the treatment of acute moderate to severe postoperative pain in adults for a maximum duration of 72 h. It is a hand-held device containing forty 15 µg sufentanil microtablets to be released directly sublingually, equal to about 2.5 mg of intravenous morphine. To prevent misuse, patients receive an individualised adhesive thumb tag to activate the SSTS with a blocking time of 20 min after each tablet. Early data showed effective treatment of acute postoperative pain with SSTS (Angelini et al. 2022) with a high degree of patient satisfaction (Donk et al. 2018; Thangaraju et al. 2023; Babazade and Turan 2016; Frampton 2016). The transmucosal application and the lipophilic properties of sufentanil ensure rapid onset of the drug. The bioavailability of sublingual sufentanil is at about 60% of intravenous application. Thus far, current studies observed a short period of SSTS postoperatively. Most studies focused on the immediate postoperative period or the overall time of hospitalisation within a typical time frame of 3 to 7 days. Therefore, there is a lack of longitudinal data evaluating the feasibility and safety of SSTS postoperatively.

Against this background, this study was conducted to assess postoperative pain control with non-invasive SSTS. Incidence of acute pain, postoperative development of POD and development of chronic pain were of interest. Therefore, our hypothesis was that SSTS is safe and can be used for treating postoperative pain.

## Methods

This prospective, clinical, longitudinal cohort phase IV trial was approved by the ethics committee of the Charité Universitätsmedizin Berlin (Ethics committee 2, Campus Charité Virchow Klinikum, EA2/041/17, Prof. Dr. jur. R. Seeland, 21.03.2017) and was registered in the study register (https://clinicaltrials.gov/ct2/show/NCT03133858). All study patients provided written informed consent to participate in the study in line with the declaration of Helsinki. Three distinct outcomes of interest were defined a priori: incidence of postoperative delirium (POD), incidence of postoperative cognitive deficit (POCD) and incidence of postoperative chronic pain (PCP). As secondary endpoints in this trial, patients were assessed for pain characterisation, depression, anxiety and stress as well as quality of life indicators. Moreover, data on patient characteristics as well as quantities of opioid requirements, side-effects and patient satisfaction was compiled. The follow-up questionnaires were also used to collect additional data on recovery after surgery and impairment of daily activities.

### Study population

We screened 127 patients receiving elective total knee arthroplasty or gynaecological abdominal surgery (i.e. laparoscopic myoma resection) between November 2017 and November 2021 at Charité University Hospital Campus Mitte or Campus Virchow Klinikum. Inclusion criteria were elective surgery and planned PCA for postoperative pain management in addition to a standardised pain medication protocol according to surgical standard operating procedures. Exclusion criteria were age < 18 years, pregnancy or nursing period, imminent emergency surgery, participation in any other clinical study during the study period and chronic opioid use for more than 3 months before the planned surgery (dose of > 20 mg/day oral morphine equivalent). Patients were closely followed and surveyed perioperatively, at 3 and at 12 months postoperatively.

### Measurement

POD was measured with the Nursing Delirium Screening Scale (Nu-DESC) (Hargrave et al. 2017) during the inpatient stay. The Nu-DESC incorporates five dimensions: disorientation, inappropriate behaviour, inappropriate communication, illusion/hallucination and psychomotor retardation. For each dimension, the examiner can give 0–2 points (0 = symptom is not observed, 1 = symptom is observed, 2 = symptom is observed strongly). It is applied by the nursing staff regularly once per shift (i.e. 3 times per day) to account for the characteristic fluctuation of POD. To define the incidence of POD, a sensitive cut-off ≥ 2 points in Nu-Desk scores was set. Postoperative chronic pain (PCP) was defined as being present if patients reported experiencing their main pain of any intensity either at least once a day or constantly after 3 months postoperatively. To identify chronic pain patients before surgery, patients were screened similarly before surgery (i.e. receiving daily oral opioids for more than 3 months). Pain intensity was measured as a numeric 11-point Likert scale and was assessed regularly on every shift during the hospital stay and on each patient visit, i.e. at least three times a day. For the analysis, we opted to use the highest pain score noted for each patient on a specific day. Side effects of pain medication and patient satisfaction with treatment and the STSS were surveyed on every patient visit. Therefore, patients were actively asked for observed side effects such as sedation, nausea and vomiting, dizziness, constipation and other unexpected effects. Patient satisfaction was measured using an 11-point Likert scale, and reasons for discomfort were assessed. In concordance with recent recommendations (Dworkin et al. 2005), further patient-related outcome measures were evaluated with a standardised assessment consisting of the EQ5D-3L with visual analogue scale (VAS) of overall health (EuroQol 2021) and a pain questionnaire adapted from the validated German Pain Questionnaire by the German pain society (Petzke et al. 2021). These were surveyed on admission to the hospital before surgery, during the inpatient stay postoperatively and at 3 and 12 months postoperatively. The EQ5D-3L is comprised of five questions regarding mobility, self-care, usual activities, pain and discomfort and anxiety or depression that can each be answered as having either no impairment, some impairment or severe impairment. The answers to these five questions can be compiled into an index value indicating the patient’s health level using a validated algorithm. The VAS is a measure of the patient’s self-reported overall health on a scale from 0 to 100 (EuroQol 2021). The pain questionnaire characterises the patient’s pain experience in more depth. It contains seven questions outlining the frequency, severity, qualities and tolerability of pain as well as the impact of chronic pain on patient’s daily lives and mental health.

### Pain treatment

All patients in this study received a non-invasive SSTS system (Zalviso®, Grünenthal GmbH Aachen) for postoperative pain management in addition to the standard of care. Orthopaedic patients received a standardised protocol of oral pain medication starting on day 0 postoperatively including 200 mg tilidine/naloxone per day, 4 g dipyrone per day, and ibuprofen 1.8 g per day. Patients undergoing gynaecological abdominal surgery received the SSTS and 4 g dipyrone as standard oral pain medication. One female additionally received an NSAID (ibuprofen), and one received a slow-release oxycodone/naloxone compound. In case of adverse effects of sufentanil or breakthrough pain, all patients could also receive, on request, individual doses of oral morphine or intravenous piritramide as rescue medication. The analgesic protocols are part of the hospital’s standard operating procedures. Some patients received spinal anaesthesia as their main anaesthesia for the respective procedure. This was performed using isobaric bupivacaine and lidocaine for local anaesthesia. Dosage was chosen depending on body weight as is standard procedure at our hospital.

### Statistical analysis

Descriptive data were summarised by mean and standard deviation or median with interquartile range (IQR) depending on scale level and data distribution. Categorical data are presented with absolute and relative numbers. Continuous data were assessed for distribution graphically and using the Kolmogorow-Smirnow test. According to data distribution and scale level, analyses of statistical significance were performed with the Wilcoxon test for dependent samples. Due to the a priori definition of three endpoints for this clinical trial, alpha was adjusted using the Bonferroni method to account for alpha-error accumulation. Therefore, statistical significance was defined with *p* ≤ 0.0167. Statistical analysis for this study was performed using IBM SPSS (Version 28.0).

## Results

**Fig. 1:**
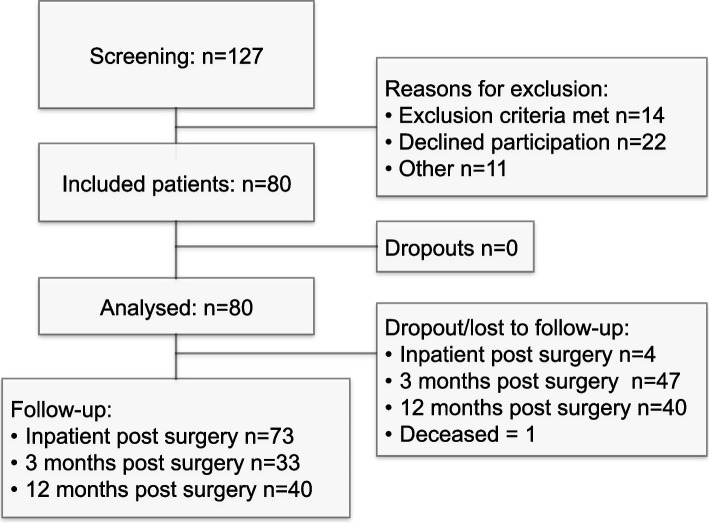
STROBE chart

After screening 127 patients, 80 patients were included in the study (Fig. 1). At 12 months, 40 patients were successfully followed up. One patient died during the study period of underlying disease non-related to the study. The baseline characteristics of the patient cohort are presented in Table 1. There were some patients who could not be reached for follow-up during the 3-month postoperative period but were successfully contacted at 12 months postoperatively. Included patients received the SSTS from day 1–3 postoperatively. The total number of retrieved sufentanil tablets differed between patients. The median sufentanil tablet dose decreased from 5 (IQR 6) on day 1 to 3 (IQR 7) on day 2 and 1 (IQR 6) on day 3 postoperatively.

**Table 1:** Baseline characteristics of 80 patients receiving the SSTS for postoperative pain-management

**Age** (in years)	
Median	67
Maximum	87
Minimum	32
**Gender** (m/f)	
Female	45 (56.3%)
Male	35 (43.8%)
**BMI (kg/m**^**2**^**)**	
Mean (± SDT)	29.1 (± 5.9)
**Surgery**	
Knee joint arthrosis	*n* = 71 (88.8%)
Abdominal surgery	*n* = 9 (11.3%)
**ASA** *(American Society of Anesthesiologists)*	
ASA I	*n* = 7 (8.8%)
ASA II	*n* = 58 (72.5%)
ASA III	*n* = 13 (16.3%)
**NYHA** *(New York Heart Association)*	
NYHA I	*n* = 44 (55%)
NYHA I	*n* = 32 (40%)
NYHA III	*n* = 1 (1.3%)
**Chronic pain-medication**	
WHO I	*n* = 20 (25%)
WHO II/III	*n* = 11 (13.8%)
**Peri-operative hypnotics**	
TIVA with propofol	*n* = 15 (18.8%)
Balanced anaesthesia	*n* = 32 (40%)
Other (including spinal anaesthesia)	*n* = 33 (41.3%)
**Intraoperative PONV-prophylaxis**	
Dexamethasone	*n* = 39 (48.8%)
Ondansetrone	*n* = 33 (41.3%)
**Intraoperative crystalloids (in ml)**	
Mean (± SDT)	1667 (± 706)
**Patients with intraoperative catecholamines**	*n* = 15 (18.8%)
**Patients with PONV**	*n* = 6 (7.5%)

### Primary endpoints

For POD, there were no patients with Nu-DESC ≥ 2 points during their inpatient stay. Thus, there was no POD detected for any patients in this study.

**Fig. 2:**
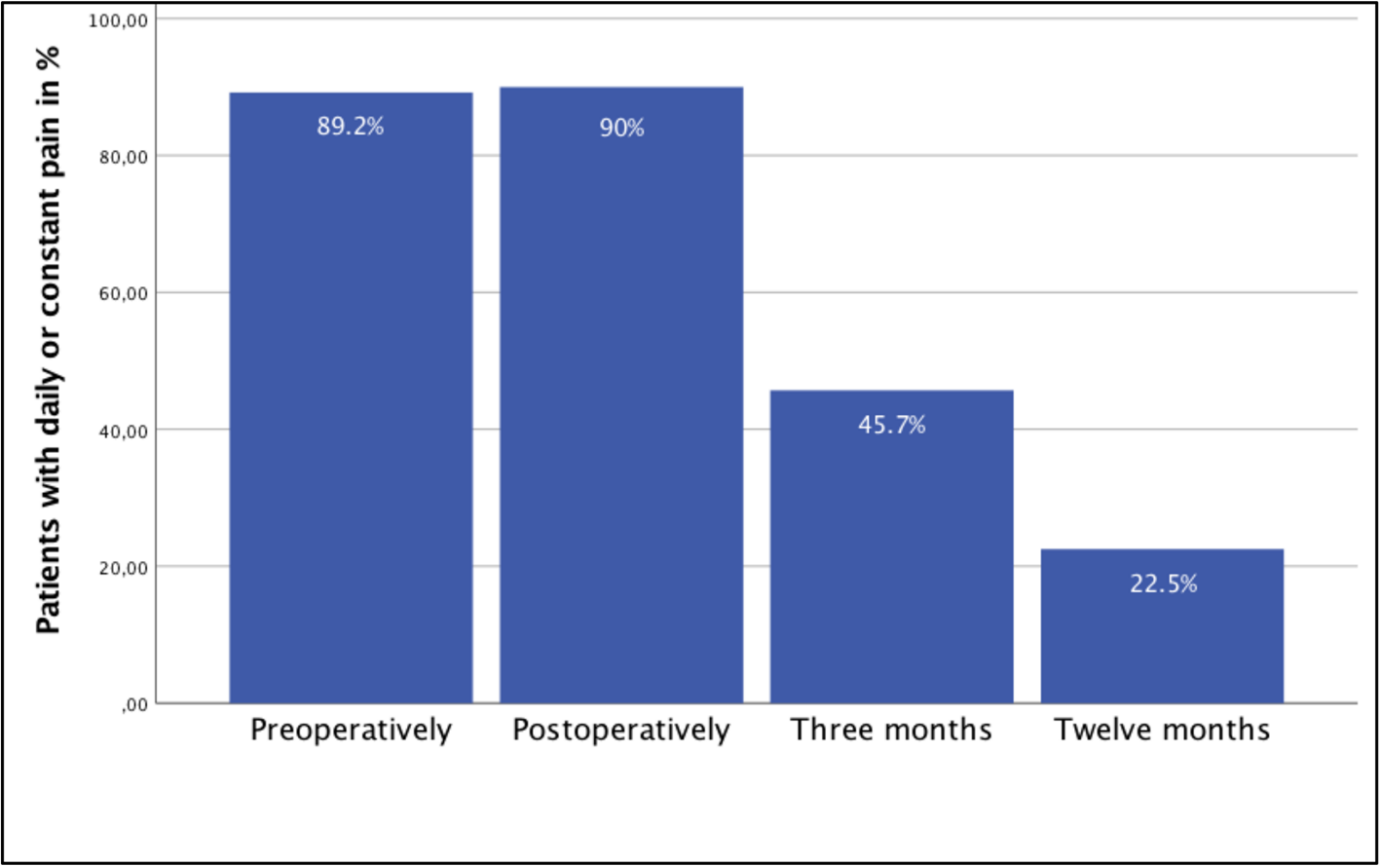
Relative number of patients who experienced their main pain either once a day, several times a day, or constantly at different times of observation

The pain questionnaire showed that 89.2% of patients experienced relevant pain (regarding the ailment of focus) *pre-operatively* either daily or constantly. During the postoperative period, 90% of patients reported pain once a day, several times a day or constantly. During follow-up, we observed an incidence of chronic postsurgical pain of 45.7% and 22.5% at 3 and 12 months, respectively (Fig. 2).

### Postoperative pain and pain management

**Fig. 3:**
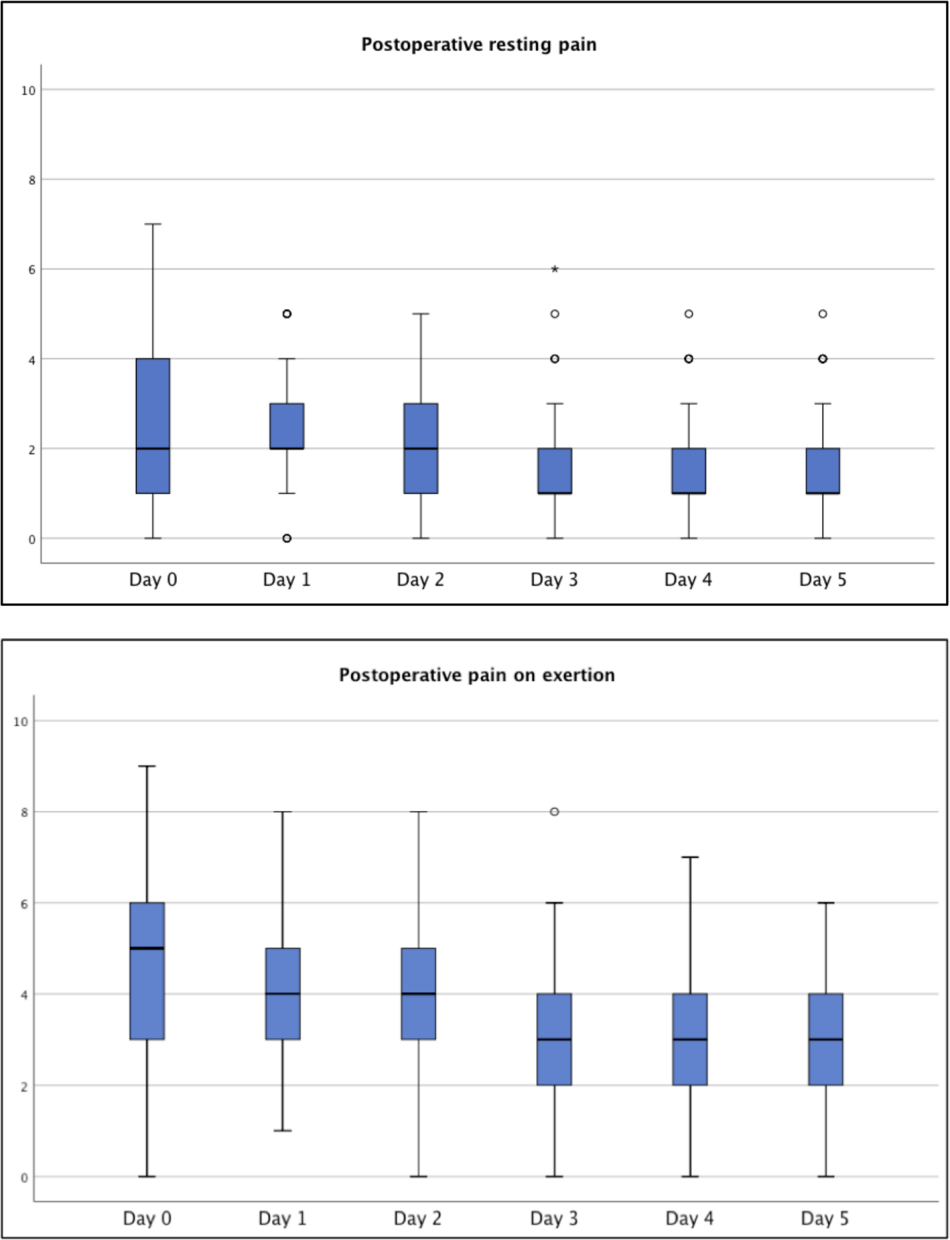
**a** Boxplot showing the distribution of postoperative resting pain in patients from the day of surgery until the fifth postoperative day. Pain is measured by the numeric rating scale (0 = no pain, 10 = strongest possible pain). **b** Boxplot showing distribution of postoperative pain on exertion in patients from the day of surgery until the fifth postoperative day. Pain is measured by a numeric rating scale (0 = no pain, 10 = strongest possible pain)

Postoperative resting pain as well as pain on exertion, measured by a numeric rating scale (NRS, 0–10) improved significantly under the treatment with SSTS and the standardised pain medication protocol from the day of surgery up to the fifth postoperative day (*p* = 0.006 and *p* < 0.001 respectively, Fig. 3a, b). Consumption of sufentanil tablets differed between patients. Overall, there was a decrease in the number of tablets used from day 1 (median 5 tbl./d) to day three (median 1 tbl./d) of the application.

**Fig. 4:**
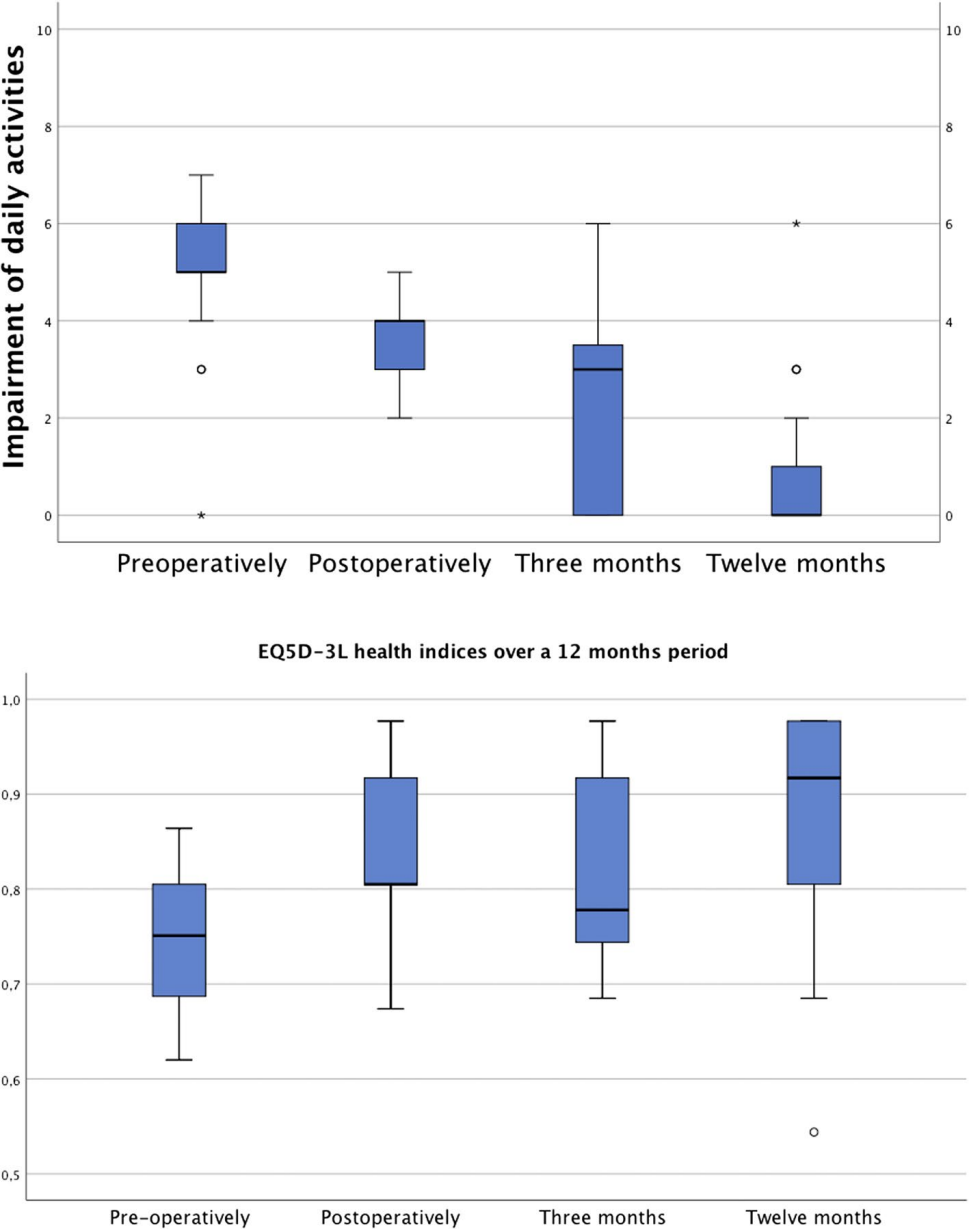
**a** Boxplot showing the improved activities of daily lives over time reported on an 11-point Likert scale (10 points representing maximum impairment of daily activities). Reported impairment of daily activities by the main pain improved significantly from pre-operatively to 12 months postoperatively (*p* < 0.001). **b** Boxplots showing the individual health indices (0–1.0) pre-operatively, postoperatively, at 3 and 12 months postoperatively

The effects of pain on patients’ daily lives decreased over time and are summarised in Fig. 4a.

### Quality of life after surgery

The individual health index measured by the EQ5D-3L improved significantly from pre-operatively to 12 months postoperatively (*p* < 0.001, Fig. 4b).

The patient’s self-reported overall health measured by the VAS also improved significantly from pre-operatively to 12 months postoperatively (*p* < 0.001). However, pain-associated emotional distress measured on a 0–10 Likert scale did not change over time (*p* = 0.421).

### Side effects and adverse events of pain treatment

No severe adverse events were observed. The most frequent unintended side effects during hospital stay were dizziness (13.8%), postoperative nausea and vomiting (PONV, 7.5%), constipation (2.5%) and other minor side effects such as temporary loss of appetite, hypotension, myosis and sedation (each 1.3%). None of the included patients had to be treated or monitored in an ICU. One patient deceased during the 1-year study follow-up period due to an underlying illness not related to this study. Notably, 51.3% of patients reported problems with the adhesive thumb tag or needed a replacement at least once during the 3-day post-operational period.

### Recovery after surgery

Altogether 73.8% (*n* = 59) of patients were mobilised to stand within 24 h postoperatively while 21.3% (*n* = 17) needed more than 24 h (Table 2).

**Table 2:** Functional parameters postoperatively during inpatient stay

**Variable**	**Median**	**Minimum/maximum**	**Interquartile range (IQR)**
Time to mobilisation (in hours h)	21h	4.5/46	7.8 h
General patient satisfaction on day of surgery (0–10 Likert scale)	8	4/10	1
General patient satisfaction day 3 post-surgery	8.5	5/10	2
Patient satisfaction day 5 post-surgery	9	5/10	2
Overall satisfaction with pain treatment	8	5/10	2
Overall satisfaction with SSTS	8	3/10	3

## Discussion

In this study, an SSTS implemented for the management of postoperative acute pain was assessed and combined with a standardised protocol of oral pain medication. The burden of chronic pain and quality of life reported by patients improved significantly at 3 and 12 months after surgery when compared to preoperative levels. Notably, POD was not detected in this study population and none of the included patients had to be treated in an ICU. The observational study design does not allow to draw any causal conclusions from these examinations.

In other publications observing POD after elective orthopaedic and abdominal surgery, the incidence of POD was described in up to 3.6–28.3% of cases (Aldecoa et al. 2017). Notably, despite fast-acting opioids being applied in this population, POD was not seen. On the other hand, this finding may be the result of unintended preventive interventions like daily study visits, the limited sample size or a specific selection of patients for study inclusion. Due to its fluctuating character, we cannot completely rule out the possibility that it was not detected despite the Nu-DESC being collected during every shift. The wide range in age may also be a factor as 50% of patients were under 67 years old. The pathogenesis of a POD is multifactorial and complex. It is impacted by a variety of factors including the postoperative pain treatment but also factors such as the kind of surgery and anaesthesia. Consistent with existing research on SSTS, our results support the notion of it sufficiently treating acute postoperative pain (Donk et al. 2018; Angelini et al. 2022; Thangaraju et al. 2023; Babazade and Turan 2016). In our cohort we found the SSTS to be a practical tool in controlling pain during early mobilisation. Moreover, most patients could be mobilised within the first 24 h postoperatively under SSTS treatment, which again underscores self-efficiency using non-invasive PCA systems. There were no severe adverse effects detected in the immediate postsurgical as well as the longitudinal setting, which can be seen as an indicator of the overall security of the device.

For the first postoperative hours, it must be stated that some patients received spinal anaesthesia, which may have had an analgesic effect for some time beyond the surgical procedure.

On the longitudinal scale, we could show that the incidence of chronic pain was substantially lower at 12 months postoperatively than pain before surgery. This should be mainly attributed to the surgical treatment of arthrosis. However, sufficient pain control with the SSTS potentially enhances recovery after surgery. The incidence of chronic postoperative pain in the literature ranges widely (Gerbershagen 2013). In Germany, where this study was performed, up to 20% of chronic pain patients state that their pain can be led back to a surgical procedure or its underlying illness (Gerbershagen 2013).

We also observed that patients’ overall health indices improved significantly by the procedure and pain treatment. Focusing simply on the perioperative stage could be too limited given the many underlying processes that cause the chronification of pain (Rosenberger and Pogatzki-Zahn 2022; Segelcke et al. 2023; Steyaert and Lavand'homme 2018). A multimodal strategy that includes psychological support and non-drug treatments like physiotherapy may be significantly more successful (Rosenberger and Pogatzki-Zahn 2022; Weinrib et al. 2017). Monitoring of patients at risk for the development of chronic postoperative pain by multidisciplinary teams during the perioperative period as well as for a few weeks following surgery might bridge the gap between immediate surgical pain care and pain management following hospital release (Rosenberger and Pogatzki-Zahn 2022). Initial observational studies of this strategy revealed decreased long-term opioid and analgesic usage (Rosenberger and Pogatzki-Zahn 2022; Glare et al. 2019).

We also observed a significant drawback during treatment with SSTS: Many patients reported issues with the authentication badge needed to allow pain medication release. Issues with hygiene were reported by some patients, as were mechanical damages on the RFID chip, e.g. gripping crutches after surgery. Therefore, many patients recommended a change of the authentication method for the SSTS device. This issue is especially important for a PCA system as its main benefit stems from being able to receive analgesic treatment on direct individual demand. Technical problems can therefore impair this benefit.

For this study, we see some limitations to be discussed. First, the study did not include a control group, which would be necessary to compare the effectiveness of the intervention itself or to draw conclusions on causality. Observational studies typically evaluate longer periods of clinical course and therefore allow the description of side effects in more real-life conditions. For comparative analyses, prospective clinical trials with randomisation would be optimal to compare the efficacy and benefits of the SSTS with other modalities. Comparative studies have been done in the past between patient-controlled intravenous analgesia and epidural anaesthesia and its effects on chronic pain (Liu et al. 2024). This could be an example of how future studies could be performed.

Selection bias is a possibility as well, as this study was not blinded due to its inherent character of testing a medical device.

In our study, we focussed on the fast-acting opioids potentially contributing to the development of POD. Since we included orthopaedic as well as gynaecological patients, we included two groups with significant proneness to the development of pain. Therefore, sensitivity analysis could not find any significant difference between the two groups.

Due to the impact of the COVID-19 pandemic especially on non-COVID-related research and restrictions on elective surgical procedures, we were not able to proceed with this trial. In addition to that the need for in-person meetings for extensive cognitive testing of patients made it impossible to collect the relevant data on postoperative cognitive dysfunction (POCD) needed for one of the three primary endpoints of this study. It might be possible that the COVID-19 pandemic has also influenced some of the results, particularly regarding self-reported overall health. The number of patients reporting an elevated risk for anxiety and stress might be at least partially explained by this. However, anxiety and distress were reported in earlier trials (Segelcke et al. 2023) as relevant contributors to patient outcomes and can be addressed (Steyaert and Lavand'homme 2018) in the hospital setting. Some of the follow-up dropout rates, which must be seen as another limitation, may be explained by these circumstances as well.

## Conclusions

This observational study expands on the existing research on perioperative pain management with PCA systems. We were able to show that PCA with sublingual sufentanil is a safe and practical tool to treat postoperative pain and observed comparable rates of PCP in patients receiving orthopaedic knee replacement or gynaecological abdominal surgery over a 12-month period. Further studies remain necessary to verify the potential advantages of the SSTS when compared to other means of patient-controlled and non-patient-controlled postoperative pain management regimens. The longitudinal approach is underrepresented in the existing research and must be further explored as well.

## Data Availability

The data supporting our research can not be made publicly available due to German data protection laws. However, certain data files can be made available by the authors upon reasonable request.

## Abbreviations

PCA: Patient controlled analgesia
SSTS: Sublingual Sufentanil Tablet System
PRO: Patient-related outcomes
PCP: Postoperative chronic pain
POD: Postoperative delirium
IQR: Interquartile range
PONV: Postoperative nausea and vomiting
RCT: Randomised controlled trial
LIA: Local infiltration analgesia
PCIA: Patient-controlled intravenous analgesia
IASP: International Association for the Study of Pain
POCD: Postoperative cognitive dysfunction
Nu-DESC: Nursing Delirium Screening Scale
NRS: Numeric Rating Scale
VAS: Visual Analogue Scale
NSAID: Non-steroidal anti-inflammatory drug
ICU: Intensive care unit
